# Endocrine-disrupting chemicals and thyroid cancer

**DOI:** 10.55730/1300-0144.6126

**Published:** 2025-10-26

**Authors:** Özge BAŞ AKSU, Mustafa ŞAHİN

**Affiliations:** Department of Endocrinology and Metabolism, Faculty of Medicine, Ankara University, Ankara, Turkiye

**Keywords:** Thyroid cancer, endocrine-disrupting chemicals, public health, pollution

## Abstract

Environmental endocrine-disrupting chemicals (EDCs) have emerged as a critical global health concern because of their role in various diseases, including thyroid cancer. Defined as exogenous substances that disrupt endocrine system functions, EDCs can affect multiple generations through mechanisms such as hormone receptor modulation, altered hormone synthesis, and epigenetic modifications.

The increasing global incidence of thyroid cancer has heightened interest in environmental factors, with EDC exposure recognized as a significant contributor. Compounds such as heavy metals, persistent organic pollutants, per- and polyfluoroalkyl substances, and bisphenol A play crucial roles in disrupting thyroid homeostasis. Emerging evidence underscores the synergistic effects of multiple EDC exposures, further amplifying cancer risk.

This review explores the relationship between EDC exposure and thyroid carcinogenesis, focusing on key chemical compounds and their mechanisms of action. Understanding these links is essential for guiding public health policies and shaping future research aimed at preventing and improving the management of this malignancy.

## 1. Introduction

Exposure to environmental pollution and its harmful consequences has become a growing global issue. In 2015, environmental pollution caused around nine million deaths globally, making it the leading cause of premature deaths, accounting for 16% of all deaths worldwide. The number represented here is three times greater than the total deaths caused by Acquired Immunodeficiency Syndrome (AIDS), tuberculosis, and malaria combined, and 15 times greater than deaths resulting from wars and violence. In countries heavily impacted by pollution, pollution-related diseases account for over 25% of all deaths [1]. According to the European Union, environmental pollutants are defined as detrimental substances introduced into the ecosystem because of human activities, and they present a risk to both human health and the environment [1]. Pollution-related chemicals, pesticides, and heavy metals contaminate air, soil, and groundwater, threatening human health, particularly in low- and middle-income countries [2]. The Lancet Commission has divided pollutants into three categories based on our knowledge of them and their effects on health. Zone 1 is the category in which pollutants are well-known and understood [1]. Endocrine-disrupting chemicals (EDCs) are part of this group.

According to a 1971 report by Herbst et al., there is evidence linking exposure to the synthetic estrogen diethylstilbestrol (DES) during pregnancy to the development of clear-cell adenocarcinoma of the vagina in young women between the ages of 15 and 22 [3]. Currently, chemicals such as DES are referred to as EDCs [4]. This early discovery laid the groundwork for understanding the broader category of EDCs, which are now recognized as significant threats to human health. As early as 2002, the World Health Organization developed a definition for EDCs [5]: “exogenous substances or mixtures that alter endocrine system functions and induce harmful effects on the health of organisms or their descendants.” EDCs interfere with hormone function from prenatal development through adulthood. The impact of these factors includes regulation of hormone receptors, hormone synthesis, hormone concentration, and hormone transport and clearance from the bloodstream via receptor-mediated binding to various cells [6]. They can modify histones and change DNA methylation, acetylation, and other epigenetic alterations [6]. However, while they can function as antagonists of endocrine nuclear receptors, they can also act as total, partial, or inverted agonists [7]. Exposure to EDCs is particularly important during sensitive periods, such as the intrauterine, childhood, and adolescent periods, when hormones have a significant impact [8]. The effects of exposure to EDCs can be observed not only in the affected individual but also in their offspring and even in future generations [9].

Understanding the dose–response features of EDCs is crucial but challenging because of the complex and variable responses of biological systems. [10]. The concentration–response curves of hormones are nonlinear, often exhibiting U- or inverted-U shapes, with the highest responses occurring at low, high, or intermittent doses. At low hormone levels, high-affinity receptor binding may occur, whereas at high levels, receptor downregulation or toxic effects may be observed. The potency or efficacy of EDCs interacting with hormone receptors is influenced by factors such as receptor isoform, abundance, and signal transduction properties [8]. Some EDCs can bind to multiple hormone receptors, making dose-dependent effects even harder to predict. Because of these complexities, accurately determining the net impacts of EDCs is difficult.

EDCs can enter the body through the skin, the gastrointestinal system, or inhalation. Plastic bottles, metal food cans, detergents, flame retardants, foods, toys, cosmetics, and pesticides are just a few of the everyday items and environmental sources that contain EDCs. Given their widespread presence, exposure to multiple EDCs is virtually inevitable. Lifelong exposure to various compounds can lead to a “cocktail effect,” resulting in a potential combination of cumulative, additive, and/or synergic effects [11,12]. Thyroid hormones are crucial for neurodevelopmental processes, metabolism regulation, and growth and development [13]. EDCs can affect thyroid functions on multiple levels and are also associated with the risk of developing functional thyroid diseases and thyroid cancers [14]. In this review, we aim to critically evaluate the influence of prenatal exposure to EDCs on thyroid hormone synthesis and function, and to explore the implications for growth, disease development, and thyroid cancer risk. The major groups of EDCs and their mechanisms affecting thyroid function and carcinogenesis are summarized in Table.

A comprehensive literature search was performed using the PubMed, Web of Science, and Scopus databases. The search focused on studies published from January 2000 to March 2024. Search terms included “endocrine-disrupting chemicals,” “EDCs,” “thyroid cancer,” “thyroid carcinoma,” “heavy metals,” “PFAS” (per- and polyfluoroalkyl substances), “BPA” (bisphenol A), and “persistent organic pollutants.” The review emphasized English-language, human-based studies, such as epidemiological research, metaanalyses, and systematic reviews examining EDC exposure and thyroid outcomes. Reference lists of relevant publications were also screened for additional sources.

## 2. Thyroid cancer and EDCs

### 2.1. Epidemiology and risk factors

Thyroid cancer is the most common endocrine malignancy, disproportionately affecting women with a female-to-male ratio of approximately 3 to 1 [15]. A combination of genetic and environmental factors contributes to the development of thyroid cancer. Genetic factors that contribute to certain types of thyroid cancer include mutations in genes like *BRAF* and *RAS*, which are often found in papillary thyroid carcinoma (PTC), as well as changes in the *RET* protooncogene associated with medullary thyroid carcinoma [16–18]. The risk of thyroid cancer increases if there is a first-degree relative (parent, sibling, or child) who has had thyroid cancer, even without a known inherited syndrome like multiple endocrine neoplasia in the family [18]. Environmental factors also have a significant impact. Several studies have consistently shown that the risk of developing thyroid cancer is significantly elevated by exposure to ionizing radiation, particularly during childhood [19]. Dietary factors, such as iodine intake, can influence the risk of developing thyroid cancer. Both insufficient and excessive iodine levels have been associated with different populations [20, 21]. Epidemiological studies suggest that over 40% of thyroid cancer cases in the United States may be linked to environmental factors such as obesity and smoking [22]. Obesity, the second most significant and modifiable risk factor for cancer after smoking, plays a crucial role in thyroid cancer development. It has been estimated that a five-point increase in body mass index and a 0.1-point increase in waist-to-hip ratio increase the risk of TC by 30% and 14%, respectively [23].

In the last half-century, there has been a notable increase in the number of thyroid cancer cases worldwide. Between 1990 and 2013, thyroid cancer’s global age-standardized incidence rate climbed by 20%. Low-income countries saw a more significant relative increase of 33%, while high-income countries experienced a 19% increase [24]. Despite its high incidence, the mortality rate of thyroid cancer remains low.

The growing number of thyroid cancers is mainly attributed to the widespread use of advanced imaging technologies, which enable us to detect small tumors more frequently and with greater accuracy. However, there has also been a rise in larger and more advanced-stage cancers, including in children [25]. Thus, it is not enough to explain the increase solely through overdiagnosis [26]. While overdiagnosis may partially explain the increase in low-risk areas, the potential contribution of environmental exposure to EDCs cannot be excluded. Given that 80% of EDCs have the potential to cause cancer, it is increasingly important to consider the link between EDCs and hormone-related neoplasias. Specifically, the connection between thyroid cancer and EDCs is critical and must not be overlooked [27]. A review analyzed studies on EDCs and endocrine tumors in humans from 1980 to 2020 [11]. EDCs were grouped based on study frequency, from the most frequently studied (pesticides) to the least frequently studied (salts). The most tumorigenic EDC groups were phthalates (phthalic acid esters, PAEs) (63%), heavy metals (54%), particulate matter (47%), and pesticides (46%) [11]. An increased risk of neoplasia was found in 43–67% of the studies. The thyroid showed the highest cancer risk (67%) following EDC exposure, suggesting a need for more detailed human studies to provide stronger evidence [11].

### 2.2. Molecular mechanisms and BRAF mutations

Thyroid-stimulating hormone (TSH) induces the proliferation of follicular thyroid cells. Even when TSH levels are within the normal range, higher levels are still associated with a greater risk of thyroid cancer [28]. TSH promotes thyroid cell growth by activating the Mitogen-Activated Protein Kinase (MAPK) pathway, where *BRAF* plays a critical role [29]. The primary oncogenic mutation in PTC, BRAF^V600E^, plays a crucial role in promoting tumor growth [30]. This mutation not only drives tumor growth but also serves as a crucial prognostic marker, linked to an aggressive tumor phenotype and an increased risk of recurrent and persistent disease [31]. It was first suggested in 2008 that there may be a relationship between the BRAF^V600E^ mutation and carcinogenic environmental exposure [32]. A high rate of BRAF^V600E^-mutated PTC has been reported in volcanic areas. This relationship may be linked to the presence of heavy metals in these regions, which represent EDCs [32].

### 2.3. Heavy metals and radioactive elements

The thyroid is more susceptible to heavy metal accumulation than other organs or tissues. This accumulation can disrupt endocrine functions [33]. Specific metal ions, such as cadmium (Cd), mercury (Hg), arsenic (As), lead (Pb), and manganese (Mn), are known to disrupt the endocrine system [33]. Chromium (Cr), arsenic (As), beryllium (Be), nickel (Ni), and cadmium (Cd) have been classified as carcinogenic substances [34]. The relationship between specific metal ions and thyroid cancer has been examined in metaanalyses. In one study, patients with thyroid cancer were found to have higher levels of copper (Cu) but lower levels of selenium (Se) and magnesium (Mn) than healthy individuals [35]. A separate study revealed that individuals with cancer had depleted levels of zinc (Zn) in their thyroid tissue [36]. Recognizing the impact of chronic, low-level metal ion exposure during childhood is crucial, as it may significantly contribute to the risk of developing thyroid cancer later in life. For instance, childhood exposure to lead (Pb) is linked to adverse health outcomes, including neuropsychological impairment [37]. A metaanalysis revealed a significant increase in manganese levels in thyroid tissue among thyroid cancer patients compared with those with benign conditions (standardized mean difference (SMD): 0.56, 95% CI: 0.16, 0.95) [38]. Conversely, cobalt levels were significantly lower in the blood of thyroid cancer patients compared with healthy controls (SMD: −2.03, 95% CI: −3.95, −0.10). There were no notable variations in the levels of other metals in the blood or thyroid tissue between those with and without thyroid cancer [38]. These findings highlight a complex relationship between metal ion exposure and the risk of developing thyroid cancer, emphasizing the need for further research into the specific roles of these ions in cancer progression.

#### 2.3.1. Uranium exposure

Uranium has 17 known isotopes, with ^234^U, ^235^U, and ^238^U being the most common in the environment because of their long half-lives [39]. All isotopes are radioactive and can cause radiotoxicity through alpha particle emissions [39]. Uranium is released into the environment through various processes, including erosion, mining, and volcanic eruptions [40]. It can enter the human body through contaminated food or water, or by inhalation, with a significant portion accumulating in bones and kidneys [39]. Studies suggest a link between uranium exposure and thyroid health issues, including an increased incidence of thyroid cancer in volcanic regions and associations with autoimmune thyroiditis observed in animal studies [39]. While the effects of radiation exposure on the thyroid gland have been extensively studied, particularly in nuclear events and radiation therapy, the impact of chronic low-level exposure to naturally occurring uranium radiation remains less understood [41,42]. The National Health and Nutrition Examination Survey (NHANES) study analyzed data to examine the link between urinary uranium levels and thyroid-related antibodies in the general population [43]. This analysis revealed a statistically significant positive association between urinary uranium levels and thyroglobulin antibody (TgAb) concentrations (p = 0.0105); however, no association was found with thyroid peroxidase antibodies (TPOab). This suggests that higher uranium exposure may be associated with increased thyroid antibody levels, which could show a risk for thyroid-related health issues. Although an ecological study did not find a significant correlation between uranium exposure and thyroid cancer at a population level, certain states with numerous uranium activity sites and high water uranium concentrations showed elevated age-adjusted thyroid cancer incidence rates [43].

### 2.4. Persistent organic pollutants (POPs) and PFAS

There is growing concern about the impact of POPs on human health and ecosystems. Common EDCs such as PFAS, polychlorinated biphenyls (PCBs), and dichlorodiphenyltrichloroethane (DDT) are prevalent in the food chain. Despite some of these chemicals being phased out since the 1970s, their long elimination half-lives have allowed them to remain significant environmental contaminants. Previous studies on the correlation between PFAS and thyroid cancer have primarily focused on highly exposed populations, often yielding controversial results [44,45]. Recent nested case-control studies have investigated this link more thoroughly, but the specific congeners associated with thyroid cancer remain unclear. The latest study aims to evaluate the association between serum levels of certain PFAS, PCBs, and DDT and the presence and characteristics of thyroid cancer in an Italian case-control cohort, marking a novel investigation in this area [46]. Notably, perfluorodecanoic acid (PFDA) levels were positively correlated with thyroid cancer (OR = 2.03, 95% CI: 1.10–3.75, p = 0.02), suggesting a significant association. The study also found a correlation between BRAF^V600E^ mutations and specific PCBs (PCB-153, PCB-138, and PCB-180). This shows a possible link between these pollutants and the molecular features of thyroid cancer. Furthermore, particular PCBs (PCB-105 and PCB-118) were linked to larger, more aggressive tumors. Interestingly, perfluorohexanesulfonic acid (PFHxS) levels showed a negative association with thyroid cancer (OR = 0.63). This negative relationship may be due to reverse causality or reflect an actual effect. In the postthyroidectomy period, metabolic rate and renal clearance increase when TSH is suppressed with levothyroxine treatment, usually at supraphysiological doses. This may have a protective effect by increasing renal clearance of EDCs, potentially contributing to reverse causality by influencing measurement methods. However, the negative association between PFHxS and thyroid cancer has been reported and emphasized in different studies [44,47,48]. Therefore, the reality of this effect remains controversial, and further research is needed to explain the underlying mechanisms.

The environmental impact of the family of PFAS remains concerning due to their persistence and bioaccumulation. Therefore, new forms of PFAS were developed to replace the old long-chain variants [49]. Although the newly developed PFAS were intended to be safer, they still have significant adverse health effects and accumulate in cells and tissues, similar to their predecessors. These new compounds are derived from the original long-chain PFAS by minor structural modifications, yielding molecules with similar industrial applications. Studies on the impact of new PFAS congeners on thyroid function show that some of these new variants exhibit similar effects. Early studies investigating the link between PFAS exposure and various cancers, though not specifically thyroid cancer, found a relatively low risk of thyroid cancer in exposed workers [49]. Therefore, while in vitro data show potential protumorigenic effects of some PFAS on thyroid cancer [50], epidemiological studies are still inconclusive. Indeed, some studies suggest a possible correlation between circulating PFAS levels and increased thyroid cancer risk [51], while others found a negative association or no association [52].

### 2.5. BPA and PAEs

BPA is an endocrine-disrupting synthetic chemical widely produced globally and found in numerous everyday items such as water bottles, food cans, dental sealants, hard plastics, epoxy resins, medical devices, baby toys, and thermal receipt paper [53]. Because of its widespread use, BPA is present in domestic environments worldwide. Exposure to BPA is a major health concern because it can disrupt endocrine signaling pathways and cause various human diseases, even at very low doses. The interaction between BPA levels, excessive iodine intake, and thyroid conditions, such as PTC and nodular goiter, has been investigated. Higher BPA concentrations were found in patients with these conditions, and multivariate analysis identified BPA and iodine as risk factors for nodular goiter and PTC [54]. The study highlights the potential interconnected pathways between BPA and iodine in the context of the pathogenesis and progression of nodular goiter and PTC [54]. Marotta et al. found that patients with thyroid nodules exposed to the BPA analog bisphenol AF (BPAF) in the presence of di(2-ethylhexyl) phthalate (DEHP) had an increased susceptibility to differentiated thyroid cancer [55]. Their combined presence led to an over 14-fold increase in the risk of differentiated thyroid cancer. This correlation was dose-independent, and the exposure to BPAF and DEHP was unrelated to higher TSH levels. The combined effects of multiple environmental pollutants, such as DEHP and BPA, may differ from exposure to a single chemical. Animal models further demonstrate that combined exposure to BPA and DEHP can significantly increase thyroid tumor susceptibility, suggesting that combined exposure to multiple EDCs may have synergistic effects. Both chemicals increased the risk of thyroid cancer by inhibiting the tumor suppressor gene *PTEN* and activating the oncogene *c-MYC* [56]. Given the potential health risks associated with BPA exposure, efforts have been made to limit its use in certain products. Many countries have implemented regulations and restrictions on the use of BPA, especially in products intended for children. However, it is crucial to continue monitoring and researching the effects of BPA to ensure consumer safety.

### 2.6. Mixture effects and population-based studies

Considering that we are not exposed to a single EDC in daily life, it is crucial to approach the investigation of the connection with thyroid cancer in this direction. Traditional methods may not accurately represent real human exposure to these chemicals. Quantile g-computation is a method for estimating the relationship between exposure to a mixture of chemicals [57]. This method is useful when working with multiple chemicals that are closely related. Recently, the first epidemiologic study focused on evaluating associations between a mix of 18 EDCs (including PCBs, flame retardants, and organochlorine pesticides) and PTC risk [58]. The study found a potentially increased risk of PTC associated with the EDC exposure mixture, but this increase was not substantial enough to be statistically significant. After stratifying by histological subtype and race, they observed an elevated risk of classical PTC among non-Hispanic white service members per one-quartile concentration increase in the EDC mixture of 18 congeners, with the top drivers of this association being PCB-180, PCB-199, and PCB-118, respectively [58]. BRAF^V600E^ mutations were linked to PCB-153, PCB-138, and PCB-180 in the recent study population [46]. The findings suggest that exposure to this EDC mixture may increase classical PTC risk, especially among white individuals, highlighting the importance of considering both racial differences and specific EDC components in understanding cancer risk.

### 2.7. Studies from Türkiye

Studies in Türkiye on EDCs and thyroid diseases have primarily focused on children and adolescents, evaluating effects on thyroid autoimmunity, function, and structure. Data connecting EDC exposure and thyroid cancer in Türkiye are limited. Existing research shows that children with total bisphenol levels above 1.3 ng/mL have about a 15-fold increase in hypothyroidism risk, with higher bisphenol levels linked to increased TSH and decreased T4 [59]. In children with Hashimoto’s thyroiditis, MEHP (a phthalate metabolite) levels are elevated, whereas superoxide dismutase, glutathione peroxidase, iodine, and zinc are reduced [60]. Negative correlations exist between BPA and fT4, and BPS and T4. Adolescents with thyroid colloid cysts have higher MEHP and mercury, increasing disease risk [61]. These findings indicate bisphenols and PAEs may contribute to hypothyroidism, Hashimoto’s thyroiditis, and thyroid cysts in children, but larger studies are needed to confirm causality.

## 3. Conclusion

This review summarizes the growing body of evidence suggesting a possible link between EDCs and thyroid cancer. Approximately two-thirds of studies have reported an increased risk following exposure, although results vary by chemical class. Polybrominated diphenyl ethers (PBDEs), PAEs, and certain heavy metals are more consistently associated with increased risk, while PCBs tend to show weaker or even negative correlations. PFAS present mixed findings. Overall, the thyroid appears to be among the more vulnerable endocrine organs to EDC-related carcinogenesis.

### 3.1. Research gaps and future directions

Mechanistic understanding of how EDCs affect thyroid cells remains limited. Both in vitro and in vivo studies are necessary to clarify the pathways involved in thyroid hormone synthesis, transport, receptor interactions, oxidative stress, genetic alterations, and epigenetic modifications. The current evidence is observational mainly, underscoring the need for long-term prospective cohorts to establish causality. Particular attention should be given to exposures during sensitive developmental windows such as childhood and adolescence.

Emerging evidence suggests that EDC-related thyroid carcinogenesis may involve interactions among multiple pathways, including oxidative stress, immune dysregulation, and epigenetic remodeling. However, most existing studies are observational, have small sample sizes, and use cross-sectional designs, which limit causal inference. Long-term prospective cohort studies that integrate biomonitoring of chemical mixtures, genotyping for susceptibility loci, and multiomics profiling of thyroid tissue would strengthen the evidence base. Additionally, standardized protocols for assessing EDC exposure and thyroid outcomes would enhance comparability across populations.

From a translational perspective, public health frameworks could benefit from focusing on three areas: biomarker discovery for early detection of exposure, regulation of industrial and food-related EDC sources, and education of healthcare professionals to identify at-risk individuals. Incorporating environmental exposure history into endocrine evaluation may improve both prevention and clinical management of thyroid disorders.

Mixture effects represent another critical gap. In daily life, individuals are exposed to multiple EDCs simultaneously, yet most studies focus on single chemicals. Advanced statistical approaches, including quantile g-computation, could be applied to evaluate potential synergistic or additive effects. The development of biomarkers to detect early exposure and identify at-risk populations would also be valuable. Furthermore, intervention studies are needed to assess whether reducing exposure leads to measurable health benefits.

### 3.2. Public health and clinical recommendations

From a public health perspective, precautionary measures are warranted. Regulatory frameworks could consider stricter limits for high-risk EDCs, alongside systematic monitoring of food and water sources. Incorporating exposure assessment into clinical guidelines would support risk evaluation in practice. Healthcare providers should be informed about potential EDC sources and equipped to advise patients on reducing exposure. Improving product labeling, promoting safer alternatives, and increasing consumer awareness are essential steps toward reducing risk.

Given their persistence and transboundary distribution, EDCs represent a global health concern. International cooperation and harmonized strategies in regulation, surveillance, and research are necessary.

### 3.3. Overall assessment

The current evidence suggests that EDCs may contribute to thyroid cancer risk, though the extent of this association varies by chemical type, exposure level, and individual susceptibility. With thyroid cancer incidence rising worldwide, coordinated efforts are needed to minimize population exposure while research continues to refine causal pathways and inform targeted prevention strategies.

## Figures and Tables

**Table:** Major groups of EDCs and their mechanisms of action on thyroid function and carcinogenesis.

EDC group	Primary examples	Main exposure sources	Mechanisms affecting thyroid function/carcinogenesis	References
**Heavy metals**	Cadmium (Cd), mercury (Hg), arsenic (As), lead (Pb), manganese (Mn), chromium (Cr), nickel (Ni), beryllium (Be)	Contaminated water, soil, volcanic emissions, industrial pollution	Accumulation in thyroid tissue, oxidative stress and direct DNA damage, disruption of thyroid hormone synthesis, interference with iodine uptake and thyroid peroxidase activity, activation of MAPK/ERK1/2 signaling, potential *BRAF* mutation induction in volcanic areas	[33–38]
**Radioactive and naturally occurring elements**	Uranium isotopes (^234^U, ^235^U, ^238^U)	Volcanic regions, mining activities, contaminated groundwater, natural erosion	Chronic low-dose radiation exposure causing DNA damage and mutagenesis, autoimmune thyroid activation with elevated thyroglobulin antibodies (TgAb), radiotoxicity through alpha particle emission	[39–43]
**POPs**	Polychlorinated biphenyls (PCBs), dichlorodiphenyltrichloroethane (DDT), dichlorodiphenyldichloroethylene (DDE)	Contaminated food chain (fish, meat), industrial waste	Disruption of thyroid hormone metabolism and receptor binding, interference with hepatic deiodinases, induction of BRAF^V600E^ mutation and association with larger or more aggressive papillary thyroid carcinomas	[44–46,58]
**PFAS**	Perfluorodecanoic acid (PFDA), perfluorooctanoic acid (PFOA), perfluorohexanesulfonic acid (PFHxS), perfluorooctanesulfonic acid (PFOS)	Nonstick cookware, food packaging, firefighting foams, drinking water	Interference with thyroid hormone transport proteins, altered TSH regulation, receptor signaling disruption, potential correlation with *BRAF* mutation, controversial evidence with both positive (PFDA) and negative (PFHxS) associations with thyroid cancer	[44–52]
**Bisphenols**	Bisphenol A (BPA), bisphenol AF (BPAF), bisphenol S (BPS), bisphenol F (BPF)	Plastic bottles, epoxy resins, dental sealants, thermal receipt paper, medical devices	Estrogen receptor agonism, inhibition of thyroid hormone synthesis and interference with iodine metabolism, oncogene activation (*c-MYC*) and tumor suppressor inhibition (*PTEN*), interaction with excessive iodine intake associated with nodular goiter and papillary thyroid carcinoma	[53–56]
